# The gustatory stalk of the Remo flounder exemplifies how complex evolutionary novelties may arise

**DOI:** 10.1038/s41598-024-55958-x

**Published:** 2024-05-22

**Authors:** Paulo Presti, Murilo N. L. Pastana, G. David Johnson, Aléssio Datovo

**Affiliations:** 1 Instituto de Biociências da Universidade de São Paulo, São Paulo, SP Brazil; 2 Museu de Zoologia da Universidade de São Paulo, São Paulo, Brazil; 3 National Museum of Natural History of the Smithsonian Institution, Washington, DC USA

**Keywords:** Ichthyology, Phylogenetics

## Abstract

The appearance of evolutionary novelties is a central issue in biology. Since Darwin’s theory, difficulties in explaining how novel intricate body parts arose have often been used by creationists and other deniers to challenge evolution. Here, we describe the gustatory stalk of the Remo flounder (*Oncopterus darwinii*), an anatomically and functionally complex organ presumably used as a chemoreceptor probe to detect prey buried in the substrate. We demonstrate that the gustatory stalk is derived from the first dorsal-fin ray, which acquired remarkable modifications in its external morphology, integument, skeleton, muscles, and nerves. Such structural innovations are echoed in both functional and ecological specializations. We reveal that the gustatory stalk arose through the gradual accumulation of changes that evolved at different levels of the phylogenetic tree of ray-finned fishes. At least five preconditions arose in nodes preceding *Oncopterus darwinii*. This finding constitutes an interesting example of how evolution can deeply remodel body parts to perform entirely new functions. In this case, a trivial support structure primitively used for swimming became a sophisticated sensory tool to uncover hidden prey.

The first known record of the Remo flounder was made by Charles Darwin during his voyage on the HMS *Beagle*. He drew attention to the presence of a “tentaculiform appendage” housed in a deep cavity on the fish's head (^1^: p. 139). However, due to poor preservation of the collected specimen, it could not be unequivocally described as a new taxon at that time (it was listed as “*Rhombus—*?”; ^1^). Formal description of *Oncopterus darwinii* came three decades later ^2^. Steindachner ^2^ illustrated the external morphology of the “tentaculiform appendage” and concluded that it was a modified first ray of the dorsal fin.

*Oncopterus darwinii* belongs to the Pleuronectiformes (Teleostei, Percomorphacea), a diverse order of marine percomorphs comprising 16 families and 820 species ^3^. Flatfish species embody one of the most peculiar morphologies in the animal kingdom ^4,5^. They have a laterally compressed body and an asymmetrical neurocranium due to the migration of an eye from one side of the head to the other, giving the adult fish one ocular (with both eyes) and one blind (without eyes) side, making them one of the most easily recognizable fish groups ^4,6–10^. The left eye is the one that migrates in *O. darwinii* and for this reason in adults both eyes lie on the right side of the body, leaving the left side without eyes (blind). *Oncopterus darwinii* is an endemic flounder of the Southwestern Atlantic Ocean traditionally allocated in Rhombosoleidae. Recent molecular phylogenies have placed the species in its own monotypic family, Oncopteridae ^11^.

Until now, no attempts have been made to study in detail the morphology and function of the supposedly highly modified dorsal-fin ray of *Oncopterus darwinii*. The present study explores the external morphology, integument, skeleton, muscles, and nerves of this structure. By comparing its morphology with that of the dorsal fin of closely related taxa, we were able to trace homologies among the structures studied and infer the evolutionary history of this organ. We also present important functional and ecological implications of the presence of this organ in *Oncopterus darwinii*.

## Results

The first modified dorsal-fin ray of *Oncopterus darwinii* is herein named as the gustatory stalk to reflect its morphological and functional singularity as a differentiated sensory structure.

### External morphology

**Figure 1:**
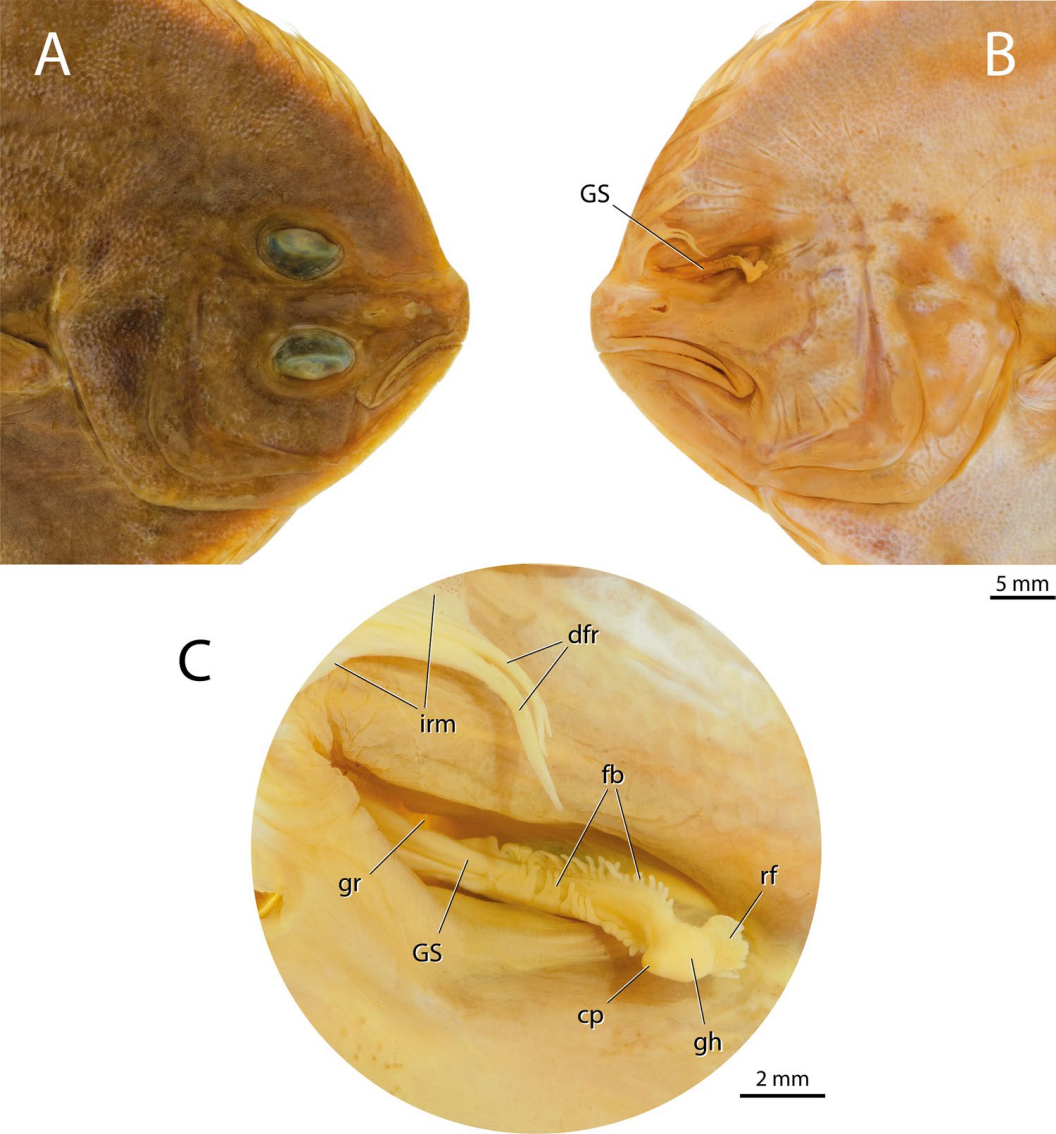
Head of *Oncopterus darwinii*; lateral views. (**A**) Ocular (right) side; (**B**) Blind (left) side; (**C**) detail of the gustatory stalk. *dfr* unmodified dorsal-fin rays; *fb* fimbriae; *gh* globular head; *gr* grove; *GS* gustatory stalk; *irm* interradial membrane; *cp* conical protuberance; *rf* rugged flap.

The gustatory stalk is accommodated into a deep groove located between the skull and the anterior portion of the dorsal-fin musculature of the blind side (Fig. 1). The organ is slightly curved laterally and much thicker and rigid than the regular rays of the dorsal fin. There is no interradial membrane connecting the gustatory stalk with the remainder of the dorsal fin. A series of fimbriae runs along the dorsal and ventral margins of the stalk. The tip of the stalk is tripartite, composed of an external conical protuberance, a central globular head, and an internal rugged flap.

### Osteology

**Figure 2:**
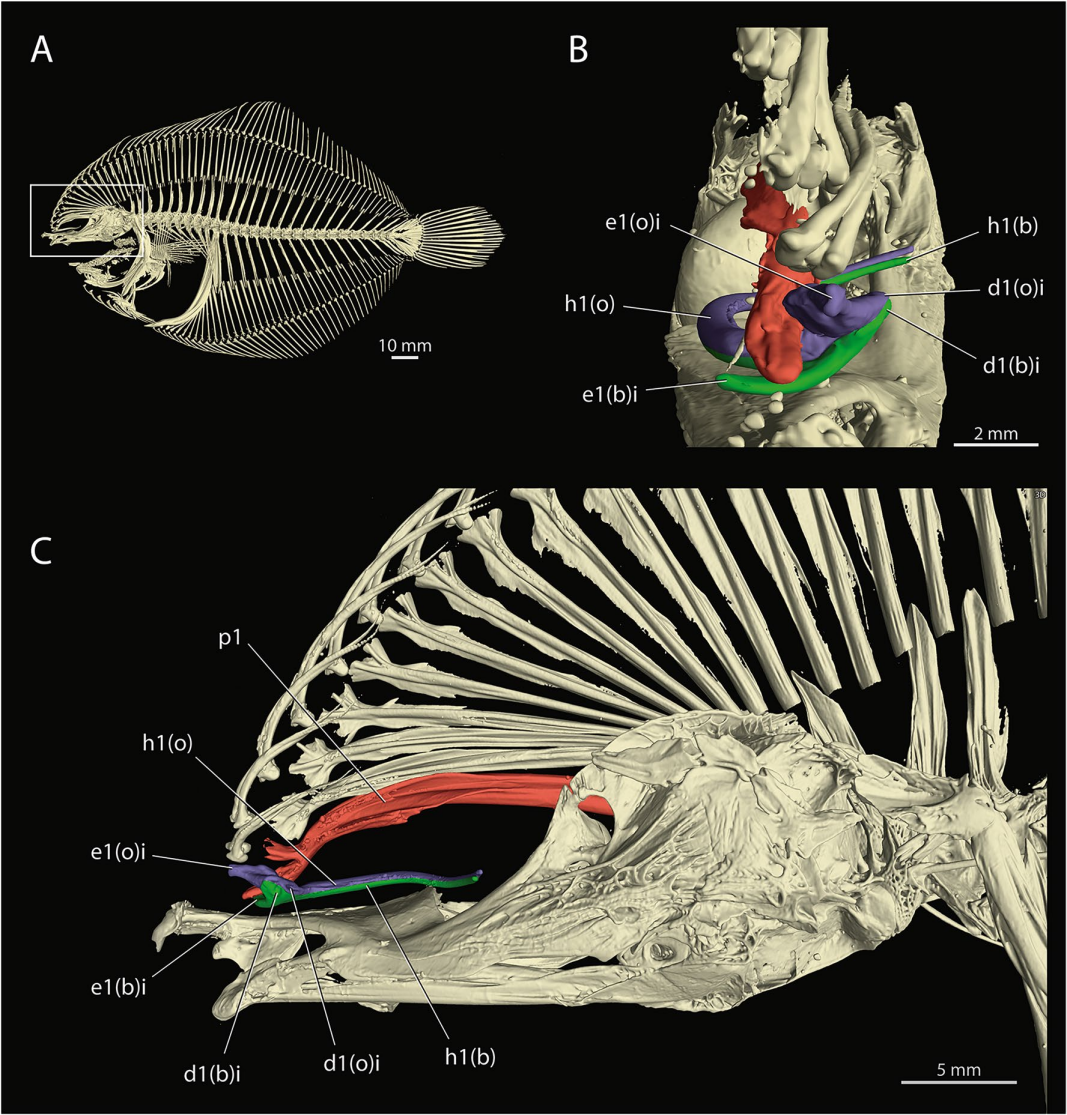
X-ray microcomputed tomographies of *Oncopterus darwinii*. (**A**) Blind side of skeleton; (**B**) Frontal view of neurocranium and associated structures; (**C**) Blind side of neurocranium. *I Ptg* first pterygiophore; *Apj* anterior projection of the gustative filament; *b* blind side (omitted when obvious); *GF* gustative filament; *o* ocular side (omitted when obvious); *Opj* ocular projection of gustative filament. Ocular-side (dorsal) hemitrichium indicated in blue; blind-side (ventral) hemitrichium indicated in green; first dorsal-fin pterygiophore indicated in red.

**Figure 3:**
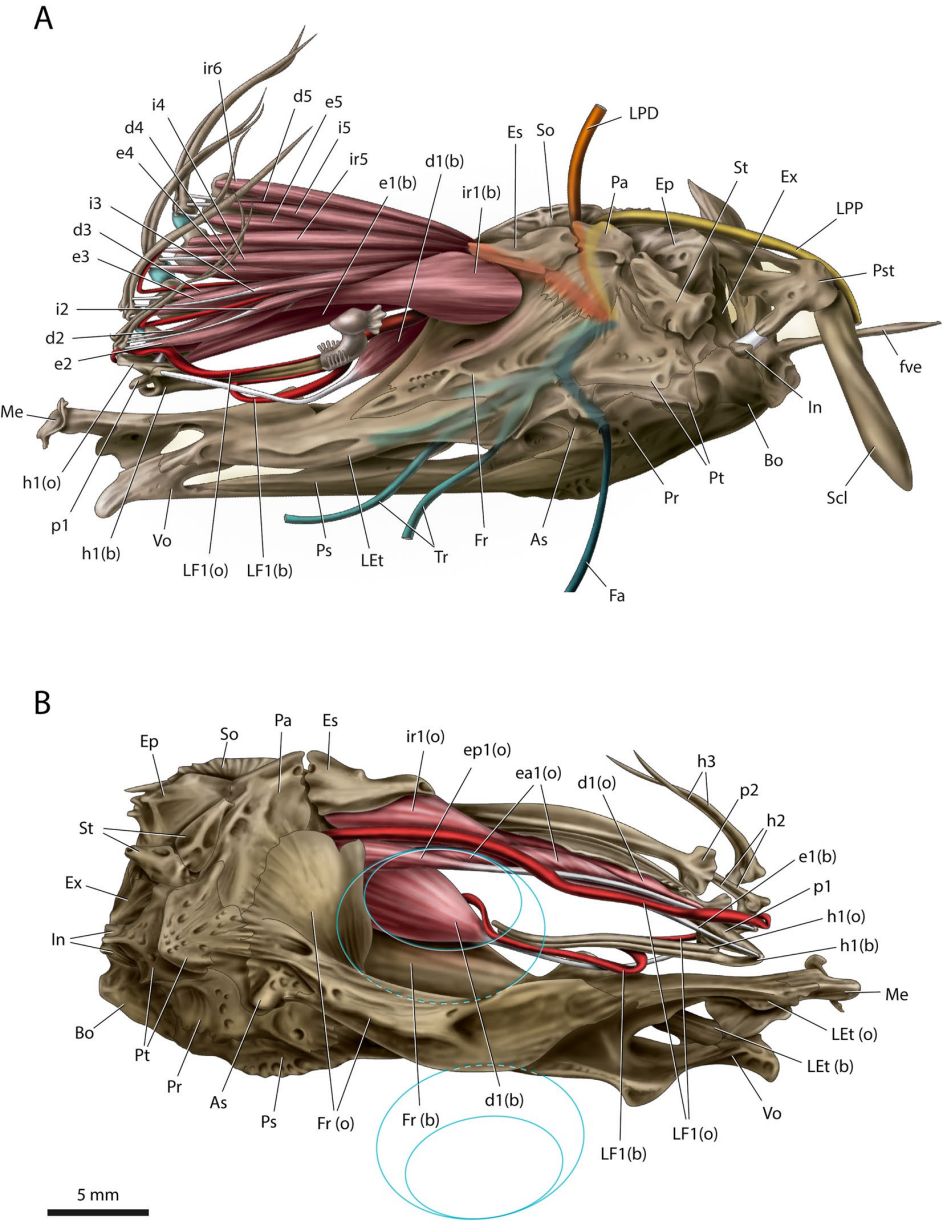
Musculoskeletal system of neurocranium, gustatory stalk, and associated structures of *Oncopterus darwinii*; lateral view. (**A**) Blind side, tip of gustatory stalk and anterior portion of unmodified dorsal fin shown; (**B**) Ocular side, tip of gustatory stalk, and muscles and nerves of anterior portion of unmodified dorsal fin omitted, blue outlines represent contour of eyes. 1–6, structures associated with dorsal-fin rays 1–6; *As* autosphenotic; *b* blind side (omitted when obvious); *Bo* basioccipital; *d* *depressor dorsalis*; *e* *erector dorsalis*; *ea* *erector dorsalis anterior*; *ep* *erector dorsalis posterior*; *Ep* epioccipital; *Es* extrascapular; *ex* Exoccipital; *Fa* facialis nerve; *Fr* frontal; *fve* first vertebra; *h* hemitrichia; *i* *inclinator dorsalis*; *In* intercalar; *ir* *inclinator dorsalis radialis*; *Let* lateral ethmoid; *LF* frontal branch of *ramus lateralis accessorius*; *LPD* parieto-dorsal branch of *ramus lateralis accessorius*; *LPP* pectoral branch of *ramus lateralis accessorius*; *Me* mesethmoid; *o* ocular side (omitted when obvious); *p* pterygiophore; *Pa* parietal; *Pr* prootic; *Ps* parasphenoid; *Pst* posttemporal; *Pt* pterotic; *Scl* supracleithrum; *So* supraoccipital; *St* supratemporal; *Tr* trigeminus nerve; *Vo* vomer.

**Figure 4:**
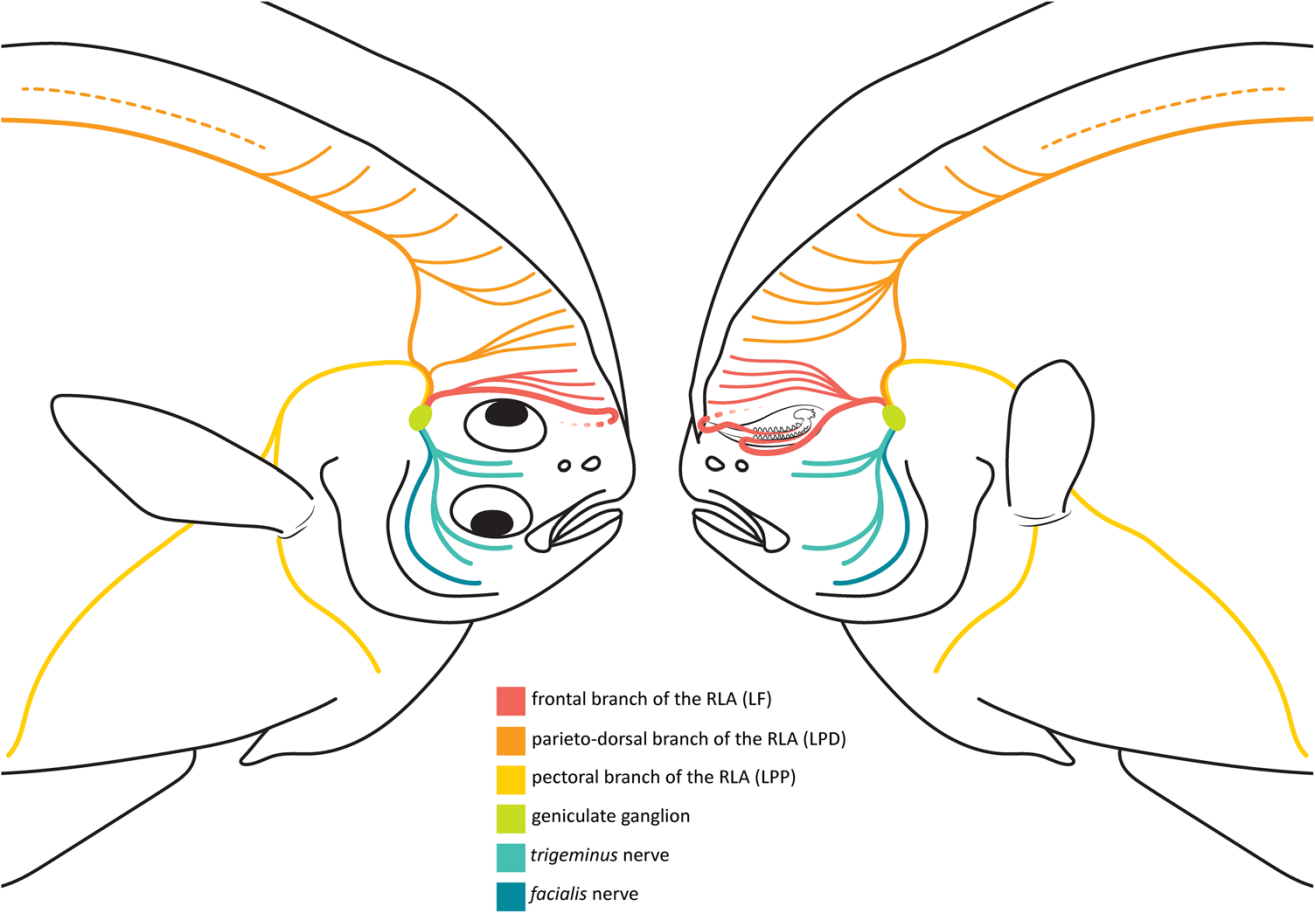
Schematic representation of the cranial nerves of *Oncopterus darwinii* associated with the geniculate ganglion.

A typical soft dorsal-fin ray in percomorph fishes is composed by a pair of segmented hemitrichium. This pattern is retained in the regular dorsal-fin rays of *Oncopterus darwinii* and, to some degree, also in its gustatory stalk. This organ is supported by the anteriormost pair of hemitrichia that are reoriented so that one is dorsal to the other (Fig. 2B,C). Muscle insertions and innervation (Figs. 3, 4) indicate that the dorsal hemitrichium is from the ocular side and the ventral one from the blind side. The bases of the hemitrichia are enlarged and have long spine-like curved projections that serve as attachment sites for dorsal-fin muscles. The dorsal hemitrichium of the gustatory stalk has a spine-like projection anteriorly oriented (anterior projection) while the ventral hemitrichium presents a spine-like projection facing the ocular side (ocular projection). The three or four subsequent rays of the dorsal fin also have the hemitrichium from the blind side slightly shifted ventrally (Figs. 2B, 3). The hemitrichia of the gustatory stalk articulates with the first dorsal-fin pterygiophore, which is distally bifurcated and more robust than the subsequent pterygiophores (Fig. 2). In resting position, the distal tip of the gustatory stalk lies dorsal to the blind-side frontal bone. The ray-pterygiophore articulation of the gustatory stalk is anterior to the migrated eye and dorsal to the lateral ethmoid and mesethmoid.

### Myology

Generally, in ray-finned fishes each dorsal-fin ray is served by three bilaterally paired muscles: *inclinatores dorsales*, *erectores dorsales,* and *depressores dorsales* (Fig. 3A)*.* The *erectores dorsales* and *depressores dorsales* usually originate from the pterygiophores and insert on the anterior and posterior regions of the base of the fin rays, respectively. The *inclinatores dorsales* usually originate from the fascia under the skin (subdermal fascia) and insert on the lateral region of the base of the hemitrichia, between the insertion sites of the *erectores dorsales* and *depressores dorsales.* Our comparative analysis shows that most pleuronectiforms have an additional muscle in at least part of their dorsal fin. This muscle inserts on the distal tip of the dorsal-fin pterygiophore and originates intercalated with the *inclinator dorsalis*. These two muscles are sometimes continuous to each other at the origin. These features suggest that the additional dorsal-fin muscle is an anterior expansion of the *inclinator dorsalis*. We then named this new muscle as *inclinator dorsalis radialis*. *Oncopterus darwinii* has *inclinatores dorsales radiales* in most unmodified dorsal-fin rays (Fig. 3), along with the regular *inclinatores dorsales*, *erectores dorsales*, and *depressores dorsales*.

The gustatory stalk of *O. darwinii* is served on each side by *erector dorsalis I*, *depressor dorsalis I*, and *inclinator dorsalis radialis I*; there is no *inclinator dorsalis I* (Fig. 3). One of the most distinctive aspects of these muscles is that they are not parallel disposed as are the muscles associated with the subsequent, unmodified dorsal-fin rays. *Inclinator dorsalis radialis I* on both sides originates from the frontal and extrascapula and inserts slightly around the midlength of the first pterygiophore. This muscle is posteriorly expanded at its origin, standing out especially on the blind side of the head. The *inclinator dorsalis radialis I* on the blind side is much enlarged compared with its ocular-side counterpart. The ocular-side *inclinator dorsalis radialis I* is partially continuous medially with *inclinator dorsalis II*.

*Erector dorsalis I* of the blind side is an elongate muscle located dorsal to the gustatory stalk, originating from the posteroventral region and from the left lateral flap of pterygiophore 1 (Figs. 2C, 3A). The muscle inserts tendinously on the ocular projection of the blind-side hemitrichium 1. The *erector dorsalis I* on the ocular side is also elongate and divided at its origin into an anterior and a posterior section that originate from the ventral region of the right lateral crest of pterygiophore 1 (Fig. 3B). Toward insertion, the two sections merge and converge to form a sturdy, elongate tendon that inserts onto the anterior projection of the ocular-side hemitrichium 1. To reach this projection, the tendon curves anteriorly towards the blind side of the head. The insertional tendons of the pair of *erectores dorsales I* cross one another near their midlength (Figs. 3B, 4).

*Depressor dorsalis I* of the blind side originates from the posteromedial face of the blind-side frontal (Fig. 3). This muscle has relatively short muscle fibers but a very long and sturdy tendon attached to the ventral tubercle of the base of the blind-side hemitrichium 1. When the gustatory stalk is adducted, this tendon is visibly loose and slightly bent. The tendon becomes strained and stretched when the gustatory stalk is manually abducted. Manipulation of the gustatory stalk indicates that it cannot be abducted anterodorsally more than about a perpendicular angle relative to the longitudinal plane, otherwise the blind-side *depressor dorsalis I* breaks down. The ocular-side *depressor dorsalis I* is extremely thin, resting in a small space between pterygiophore 1 and the ocular parts of *erector dorsalis I* and *inclinator dorsalis radialis I*. *Depressor dorsalis I* originates from the dorsal region of the right lateral crest and inner region of the right lateral flap of pterygiophore 1. The insertion is tendinous on a tubercle at the base of the ocular-side hemitrichium 1, immediately dorsal to the insertion site of the blind-side *depressor dorsalis I* (Fig. 2B). To reach this site, the insertional tendon passes between the distal bifurcation of pterygiophore 1.

### Neurology

The *ramus lateralis accessorius* (RLA) is a paired nerve that carries only gustatory fibers that innervates taste buds located outside the mouth (= terminal buds) in fishes ^12,13^. The RLA in *O. darwinii* emerges dorsally from the geniculate ganglion and, before exiting the braincase, bifurcates into an anterior (frontal) and a posterior (parietal) branch (Fig. 4). The anterior branch, herein termed frontal branch of the *ramus lateralis accessorius* (LF), exits the neurocranium through a foramen in the frontal and bifurcates into a dorsal and a ventral *ramulus*. The ventral *ramulus* (LF1) is thicker and serves exclusively the gustatory stalk (Fig. 3), whereas the dorsal one is thinner and innervates the immediately subsequent dorsal-fin rays.

LF1 of the ocular side runs anteriorly toward the bifurcation of pterygiophore 1 and then curves to the blind side to reach the base of the ocular-side hemitrichium 1 (Fig. 3). It then parallels the entire outer border of this hemitrichium. The blind-side LF1 runs straight toward the gustatory stalk after its exit from the braincase. However, this branch makes a sharp U-shaped bend before reaching the ray’s base and paralleling the outer border of the distal two-thirds of the blind-side hemitrichium 1.

The dorsal *ramulus* of the RLA of the ocular side gives off branches to the ocular-side hemitrichium of dorsal-fin rays 2–4 (Fig. 4). On the blind (left) side, this *ramulus* innervates the blind-side hemitrichium of dorsal-fin rays 2–5. The posterior (parietal) branch of the RLA exits the neurocranium from the parietal and runs posterodorsally to the neurocranium. This branch then divides into a parieto-dorsal (LPD) and a pectoral subbranch (LPP; Figs. 3, 4). The former innervates the remaining dorsal-fin rays and the latter the pectoral, pelvic, and anal fins.

### Integument

The integument of the gustatory stalk exhibits Types I and II taste buds (sensu Reutter, et al. ^14^) scattered along the central axis, the marginal fimbriae, and the internal rugged flap. Type I taste buds are more frequent on the distal tip and Type II buds are more frequent on the central axis. The fimbriae house both types of taste buds.

**Figure 5:**
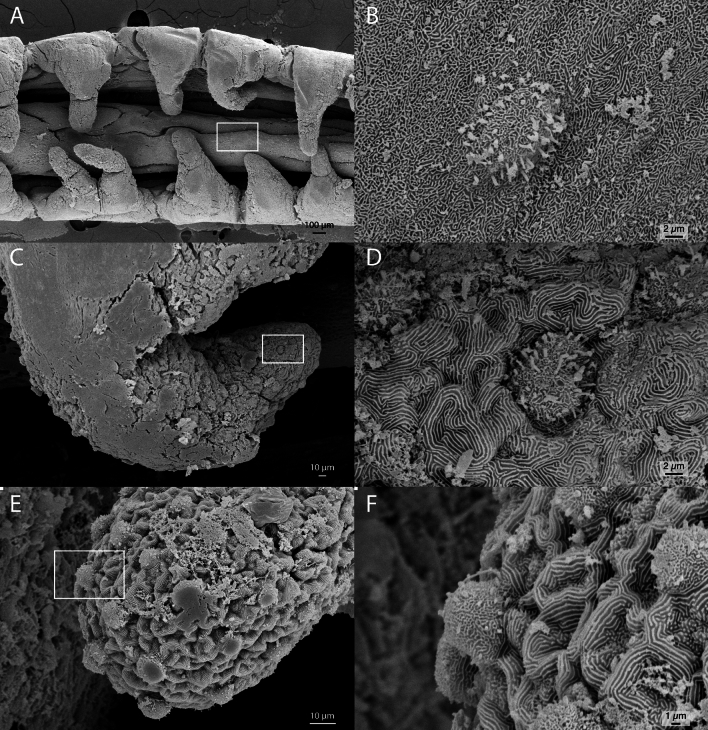
Scanning electron microscopy of the gustatory stalk of *Oncopterus darwinii*. (**A**) Part of external (left) surface showing its main axis and marginal fimbriae. (**B**) Zoom-in of the rectangle on image A showing a type II taste bud surrounded by microridges. (**C**) and (**E**) Zoom-in of fimbria with several taste buds on their surfaces. (**D**) Zoom-in of the rectangle on image (**C**) showing a type I taste bud surrounded by convoluted microridges. (**F**) Zoom-in of the rectangle on image (**E**) showing a type II taste bud surrounded by convoluted microridges.

**Figure 6:**
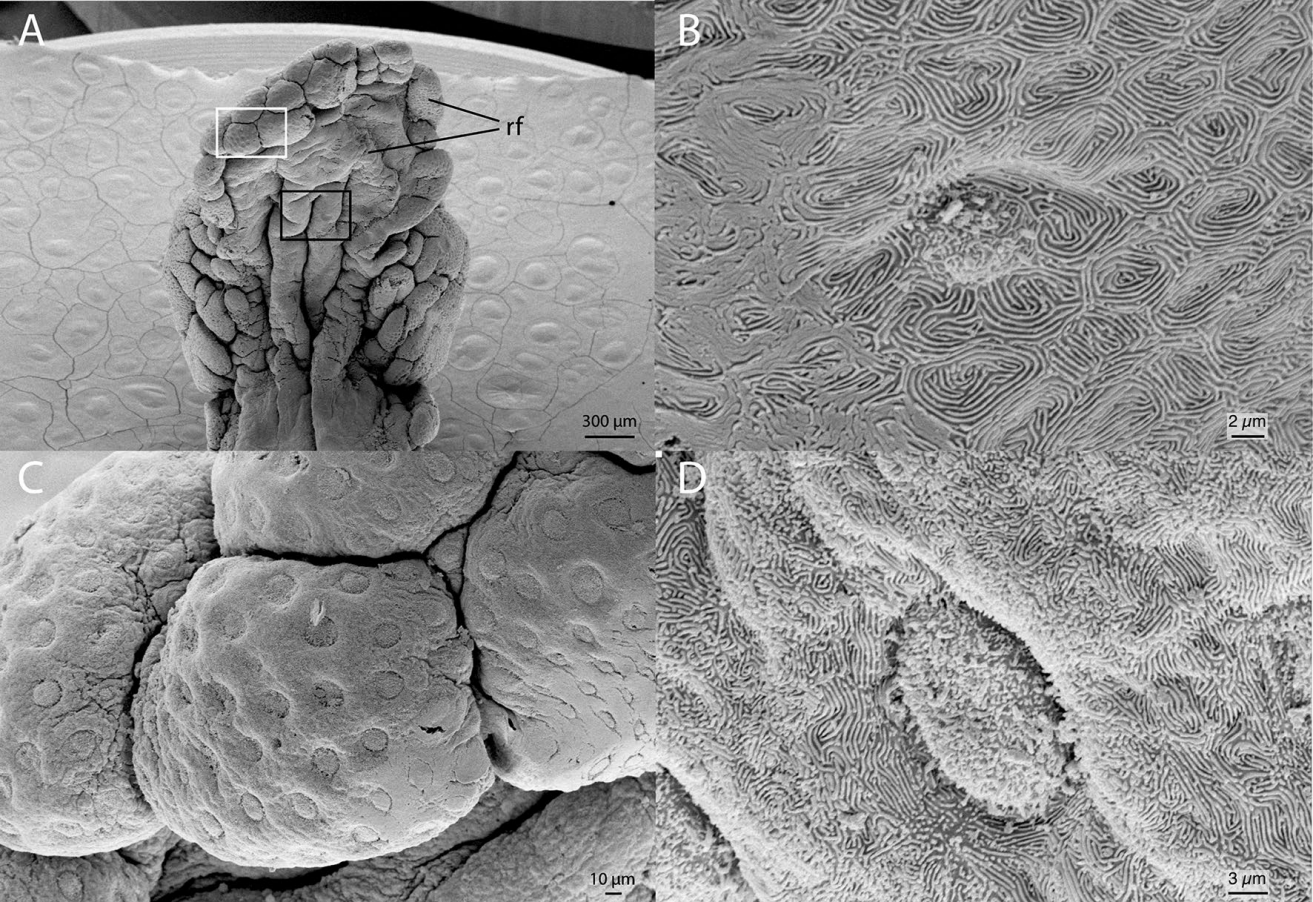
Scanning electron microscopy of the globular head of the gustatory stalk of *Oncopterus darwinii*. (**A**) Medial (internal) view. (**B**) Zoom-in of the black rectangle on (**A**) showing a type I taste bud surrounded by microridges. (**C**) Zoom-in of the white rectangle on (**A**) showing several taste buds. (**D**) Zoom-in of the rectangle on image A showing a type I taste bud surrounded by microridges. *rf* rugged flap.

Taste buds on the main axis are sparsely distributed and surrounded by shallow microridges that do not form well-defined pathways (Fig. 5A,B). In contrast, taste buds on the fimbriae and on the rugged flap are more densely distributed (Figs. 5C–F; 6C). The former are surrounded by well-developed microridges over an uneven surface that form labyrinthine pathways (Fig. 5D,F).

**Figure 7:**
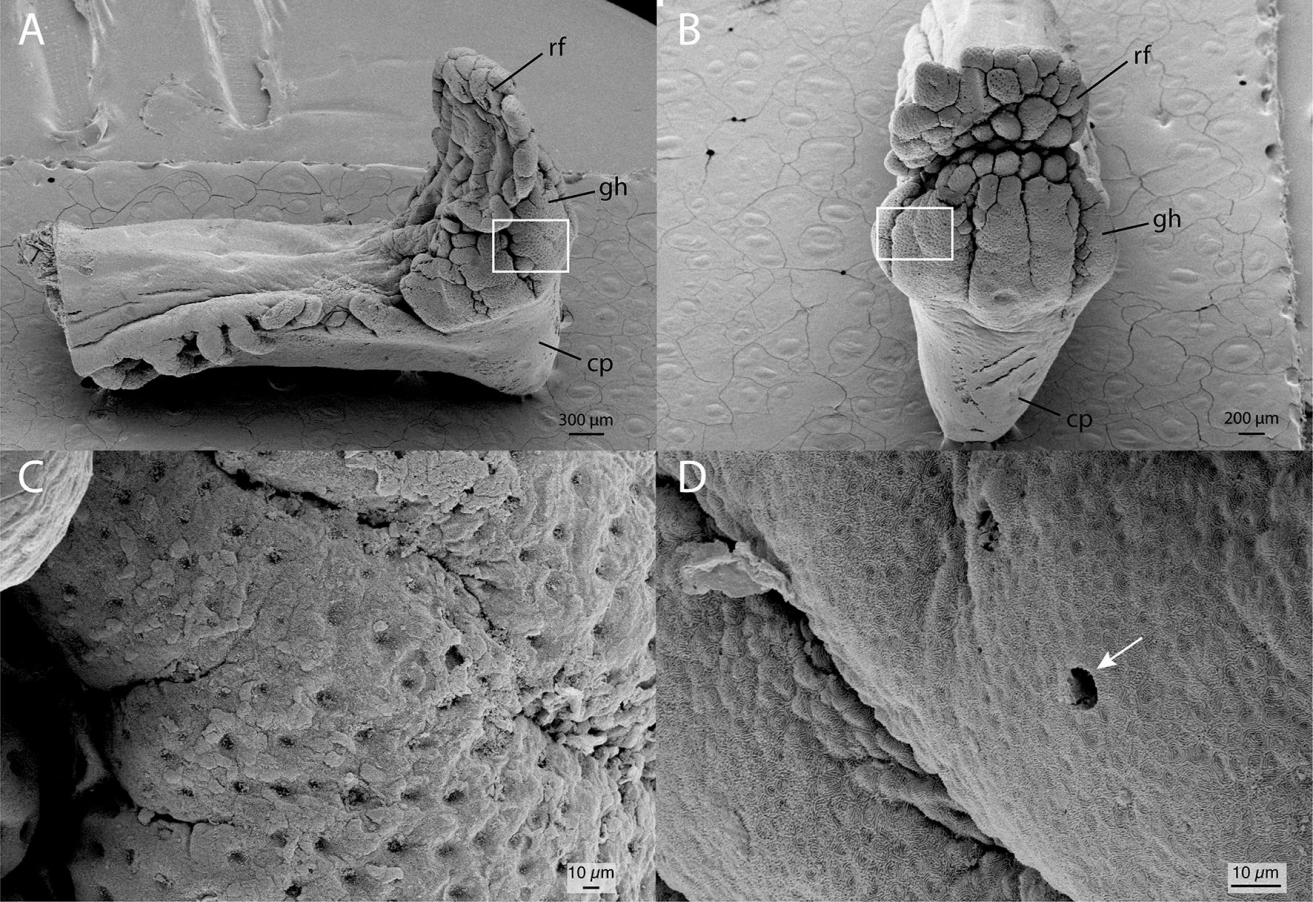
Scanning electron microscopy of the distal tip of the gustatory stalk of *Oncopterus darwinii*. (**A**) Dorsal view. (**B**) Left lateral view. (**C**) Zoom-in of the rectangle on images A and B showing numerous dimples on the surface of the globular head. (**D**) Zoom-in of the rectangle on images A and B, arrow indicates a goblet cell opening. *gh* globular head; *cp* conical protuberance; *rf* rugged flap.

The external conical protuberance of the distal tip has no taste buds, nor does the globular head, which is covered by innumerous dimples (Fig. 7A–C). The rugged flap is marginally covered by numerous closely spaced Type I taste buds (Fig. 6A,C,D). The inner region of the rugged flap is rippled and also shows Type I taste buds (Fig. 6B). The taste buds of both the inner surface and marginal regions of the rugged flap are surrounded by microridges that form well-defined labyrinthine pathways.

Goblet cells are widely distributed throughout the gustatory organ, mainly on the dorsal and ventral surfaces of its main axis and on the inner surface of the distal tip (Fig. 7D). No distinct pattern of goblet cell distribution was evident.

**Figure 8:**
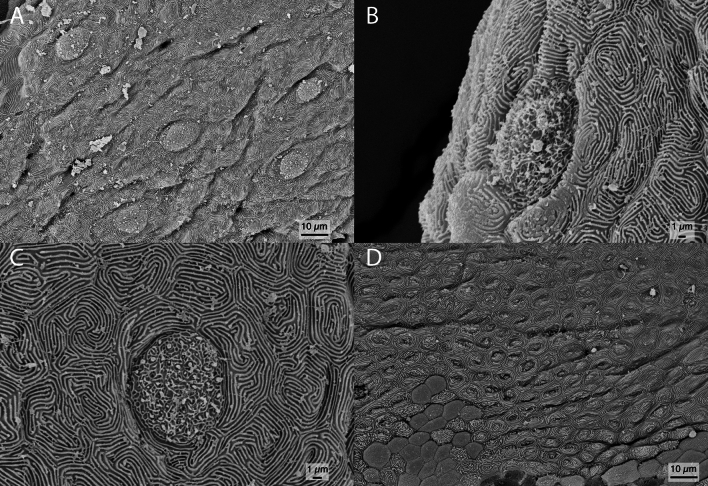
Scanning electron microscopy of the unmodified dorsal-fin rays of *Oncopterus darwinii*. (**A**) Tegument of the second dorsal-fin ray with taste buds. (**B**) Zoom-in of a taste bud from the second dorsal-fin ray. (**C**) Zoom-in of a taste bud from the 31st dorsal-fin ray. (**D**) Tegument of the 55th dorsal-fin ray without taste buds.

The remaining dorsal-fin rays of *O. darwinii* exhibit only Type I taste buds (Fig. 8A–C). The concentration of taste buds gradually decreases posteriorly, but taste buds are still present posteriorly to at least the 31^st^ dorsal-fin ray (Fig. 8C). The overall shape of these taste buds resemble those found on the gustatory stalk. Microridges are also present around each taste bud. The posteriormost dorsal-fin rays lack taste buds (Fig. 8D), and we could not determine exactly from which ray the taste buds cease to exist.

## Discussion

Although Darwin left unexplained the homology of the gustatory stalk (his “tentaculiform appendage”; ^1^) of the Remo flounder, Steindachner ^2^ correctly concluded that it corresponded to a highly modified first ray of the dorsal fin. This interpretation has been followed by all subsequent authors ^11,15^, but no study until now has provided evidence to support this claim. The osteological and myological evidence collected here clearly shows that the gustatory stalk is homologous to the first dorsal-fin ray of other fishes: the stem of the organ is supported by the anteriormost pair of hemitrichia articulated with an expanded first pterygiophore and receives three sets of bilaterally paired muscles, the *erector dorsalis I, depressor dorsalis I,* and *inclinator dorsalis radialis I*.

However, the organ has so many morphological and functional modifications compared to an unmodified dorsal-fin ray that one naturally wonders how such a structure could have evolved. A comparative analysis reveals that several changes accumulated gradually during the evolution of the lineages leading to *O. darwinii*, with several preconditions arising at different levels of the phylogeny of ray-finned fishes.

Two preconditions probably preceded the origin of the flatfishes (Pleuronectiformes), although it is difficult to precisely pinpoint the phylogenetic nodes at which they appeared due to lack of a consensus on the sister group of flatfishes and because soft tissues are generally poorly studied in extant fishes rarely preserved in fossils. One of the first preconditions to evolve was the presence of taste buds innervated by the *ramus lateralis accessorius* (RLA) in the dorsal fin. This condition has been reported in other flatfishes (e.g., Livingston ^16^) and we additionally found it in *Ammotretis rostratus*. Although information on dorsal-fin taste buds and RLA innervation is relatively scarce in the literature, they are known to be present in many (possibly most) teleost lineages ^12,13^, suggesting that they may have a quite ancient origin. *Oncopterus darwinii* then simply developed a gustatory stalk with a much higher concentration of taste buds (Figs. 5, 6, 7, 8) that were already primitively present in an ancestor.

The dorsal fin extending anteriorly to the neurocranium is a precondition for the origin from the neurocranium of part of the musculature serving the gustatory stalk. Such an anteriorly extended dorsal fin is present in all flatfishes, as well as in several other percomorph groups, such as stromateiforms, caristiids, some scorpaeniforms, and several carangoids ^17^. Although there is no consensus on the sister group of flatfishes (cf. ^18–22^) they are often resolved as closely related to carangoids. This raises the possibility that this trait arose in an ancestor common to both clades, with inevitable reversals occurring in some subgroups.

Two preconditions for the gustatory stalk unequivocally evolved at the base of Pleuronectiformes. First, the notorious bilateral asymmetry of the eyes and other structures in adults. Bilateral asymmetry in adults is not limited to the head and includes modifications in body pigmentation, paired fins morphology, swimming behavior, and different muscle sizes between ocular and blind sides, for example ^6,23^. This asymmetry is further coupled with a unique benthic behavior in which the blind side of the body is placed against the substrate. As a result, the dorsal fin of flatfishes is almost constantly in contact with the substrate, a condition unparalleled in any other living or extinct fishes. It is difficult to imagine another group of fishes in which a sensory organ for digging into the substrate could have evolved from the dorsal fin, since in other fishes this fin usually never touches the bottom.

**Figure 9:**
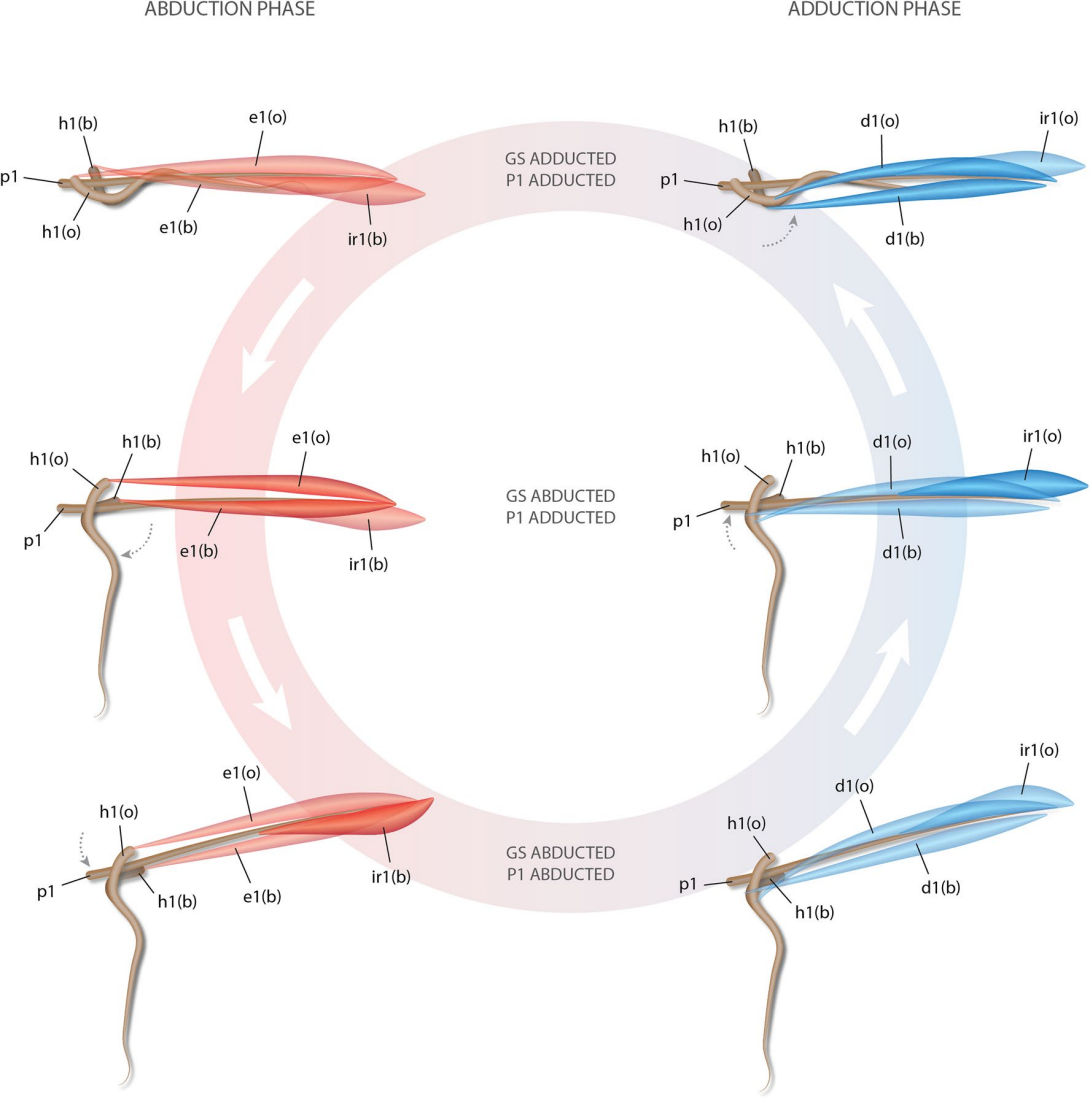
Schematic representation of the inferred biomechanics of the musculoskeletal system of the gustatory stalk of *Oncopterus darwinii*. *b* blind side; *d* *depressor dorsalis*; *e* *erector dorsalis*; *GS* gustatory stalk; *h* hemitrichia; *i* *inclinator dorsalis*; *ir* *inclinator dorsalis radialis*; *o* ocular side; *p* pterygiophore. Muscles active during abduction and adduction phases shown in red and blue, respectively; muscles promoting each movement shown in more intense colors.

The *inclinatores dorsales radiales* are specialized muscles found in the dorsal-fin rays of *Psettodes* and most pleuronectoids we examined. To our knowledge, these muscles are not found elsewhere in ray-finned fishes (cf.^24^). They appear to be anterior expansions of the regular *inclinatores dorsales* that have acquired novel insertions at the distal tip of the dorsal-fin pterygiophores (see Results: Myology). Based on their attachments, contraction of the *inclinatores dorsales radiales* probably moves the base of the dorsal fin of pleuronectiforms. While *inclinatores dorsales radiales I* seem to dig the abducted gustatory stalk further into the substrate (Fig. 9), in the remaining rays of *Oncopterus darwinii* and other flatfishes these muscles seem to act in locomotion by undulating the base of the dorsal fin—and thus the dorsal edge of the trunk. Not surprisingly, corresponding muscles are often found in the anal fin, the *inclinatores anales radiales*, which attach to the distal portion of the anal-fin pterygiophores. Undulation of not only the dorsal and anal fins but also their bases is important for locomotion in many flatfishes ^25,26^.

One last precondition related to the asymmetry of the gustatory stalk has evolved in nodes within Pleuronectiformes. The anteriormost dorsal-fin rays directed to the blind side, twisting their orientation so that the ocular- and blind-side hemitrichia acquire dorsal and ventral positions, respectively. This condition is present in most pleuronectoids examined, being absent in *Psettodes* and all non-flatfish percomorphs, thus being optimized as having appeared in Pleuronectoidei with reversals in Achiridae, Cynoglossidae, and Soleidae.

Dorsal and anal fins of Pleuronectiformes play an important role in the lifestyle of flatfishes, which can be used to display complex behaviors, such as: swimming in open water, body clamping in order to remain steady on the seafloor, burying, and even “walking” on the substrate through anteroposterior ripple movements of the soft rays ^10,25,27^. Moreover, the pleuronectiform dorsal fin can further influence food intake dynamics, as seen in the Ambush predator angler flatfish (*Asterorhombus fijiensis*) ^28^. In this taxon, the first dorsal-fin ray is modified into an elongated membranous lure that resembles small fishes or crustaceans, and is used to attract prey ^10,28^. Rhombosoleids such as the New Zealand sole (*Peltorhamphus novaezeelandiae)*, Yellowbelly flounder (*Rhombosolea leporina)*, New Zealand sand flounder (*R. plebeia)* ^16^, and Longsnout flounder (*Ammotretis rostratus*—*pers. obs.*) also show interesting modifications on their dorsal-fin rays that are related to food intake. According to Livingston ^16^, the first 16–17 dorsal-fin rays of *P. novaezeelandiae* are free and have a cup-shaped dermal outgrowths on the front of each ray. These cup-shaped structures are filled with taste buds, which are used as a sensory organ for searching for food within the sand. *Rhombosolea leporina* and *R. plebeia* also present taste buds distributed throughout their dorsal-fin rays ^16^. *Ammotretis rostratus* has several protuberances dispersed along the dorsal-fin rays that carries taste buds. Livingston ^16^ also observed in experiments conducted in aquaria, that some rhombosoleids also move their dorsal-fin rays filled with taste buds back and forth across the sand. Livingston ^29^ further analyzed the diets of these species and found that these fishes are specialized in capturing benthic infauna.

Ecological evidence is also consistent with the hypothesis that the gustatory stalk is a sensory organ used to detect prey buried in the superficial layers of the substrate. *Oncopterus darwinii* is a demersal species commonly found in sandy bottoms, a soft substrate that could be easily penetrated by the gustatory stalk ^30–32^. Available information on the diet of the species indicates that it feeds heavily on *Bathyporeiapus bisetosus* (88–92% frequency and 73–87% index of relative importance) and *Emerita brasiliensis* (19–28% frequency and 4–6% index of relative importance; ^31^, both macrobenthic interstitial crustaceans ^33,34^. Another important item in their diet were mysid crustaceans (19–57% frequency and 7–28% index of relative importance; ^31^). Although not identified to species level in the diet study, most mysids are known to live on or buried in the substrate ^35^. Therefore, interstitial prey has an index of relative importance of 77–99% in the diet of *O. darwinii*.

We provide compelling evidence that this modified first dorsal-fin ray of *O. darwinii* is capable of tasting. The organ is covered by two types of taste buds and is innervated by thick branches of the *ramus lateralis accessorius* (RLA), a nerve that invariably carries gustatory fibers ^13^. Norman ^15^ previously suggested that the organ might “be sensory”, without providing evidence or mentioning which senses might be involved.

We found many taste buds of Types I and II (sensu ^14^) in the integument of the gustatory stalk. Both types have chemoreceptive and mechanoreceptive capacities ^14,36–38^. Goblet cells are also distributed throughout the integument. These cells produce mucus, which has protective functions such as lubricating the epithelium against abrasion, protecting it from mechanical injury, and inhibiting the proliferation of some pathogens ^37–39^. Goblet cells and taste buds are extensively surrounded by microridges. These convoluted structures are known to increase the retention of protective mucus over the epithelium ^14,40–42^. The microridges are more developed and form deep labyrinthine pathways in the fimbriae and the marginal region of the rugged flap. Not coincidentally, these regions are the most delicate and have a higher density of taste buds – and thus are the most sensitive parts – of the gustatory stalk.

The nerves serving the gustatory stalk and other dorsal-fin rays of *O. darwinii* meet all the criteria proposed by Freihofer ^13^ for their identification as branches of RLA. They arise from the geniculate ganglion, from which the *trigeminus* and *facialis* nerves also diverge. All RLA branches then run peripherally to innervate the numerous taste buds located on the integument of the dorsal-fin rays. Finally, most RLA branches follow trajectories similar to those described in other percomorphs ^13^.

The presence and distribution of the RLA in other flatfishes is controversial. The nerve has been reported to be absent in some taxa ^13^ but present in others ^43,44^. Sato, et al. ^45^ recently questioned the presence of the RLA in flatfishes, arguing that previous reports misidentified a branch of a lateral-line nerve (the dorsal ramule of superficial ophthalmic ramus; SORd) as the RLA. While such misidentification may have occurred in some previous studies, this is definitely not the case in *O. darwinii* and probably not in some other flatfishes. While the SORd terminates in a series of superficial neuromasts located between the dorsal margin of the neurocranium and base of the dorsal fin, the branches of the RLA in *O. darwinii* extend dorsally into the fin and run parallel along the entire length of the rays (Fig. 3). Moreover, the RLA is the only nerve known to innervate taste buds outside the oral cavity ^13^. Other flatfishes are known to have taste buds on the dorsal fin and therefore probably also have RLA (e.g., *Peltorhamphus novaezeelandiae*, *Rhombosolea leporina*, *R. plebeia*; ^16^). Although we did not examine neuromast innervation in our study, we identified a small branch from the *trigeminus in O. darwinii* that appears to correspond to the SORd of Sato, et al. ^45^.

In *O. darwinii*, the RLA exits the geniculate ganglion and subdivides into branches that run throughout the body. The anteriormost *ramus* slightly resembles the orbito-pectoral branch (RLA-OP) described by Freihofer ^13^ for other percomorphs in that it is the only branch that is anteriorly directed after leaving the braincase. However, it does not emerge from the trigeminal foramen, curve posteriorly, or innervate the pectoral fin as does the RLA-OP of percomorphs. Rather, this anterior RLA branch in *O. darwinii* exits the braincase from the frontal, runs toward the anterodorsal region of the head, and innervates the anteriormost dorsal-fin rays. Such a condition differs from all 16 RLA patterns documented by Freihofer ^13^. Therefore, we are herein naming this branch as the frontal branch of the *ramus lateralis accessorius* (LF). The parieto-dorsal and pectoral branches (LPD and LPP, respectively) of *O. darwinii* are similar to Pattern 7 of Freihofer ^13^, reaching the posteriormost dorsal-fin rays, anal fin, and pectoral and pelvic girdles.

The gustatory stalk has many musculoskeletal changes compared to the remaining rays of the dorsal fin. First, the muscles serving the organ are much larger than those associated with the unmodified dorsal-fin rays (Fig. 3). Also, the lepidotrichia of the gustatory stalk are rearranged to lie completely over the blind side of the body (Fig. 1). In addition, the hemitrichia are twisted so that the ocular-side one assumes a dorsal position while the blind-side one becomes ventral (Fig. 2). As a result, the spinous processes for insertion of the pair of *erectores dorsales I* are directed to the ocular side and the tubercles for insertion of the *depressores dorsales I* are directed to the blind side. Despite the rearrangement on the hemitrichia of the gustatory stalk, the *erectores dorsales I* and *depressores dorsales I* retain their origin on each side of the body (Fig. 3). As a result, the *erector dorsalis I* of the blind side invades the ocular side to reach its insertion site; the opposite is true for the ocular-side *depressor dorsalis I*.

Based on the attachment sites and manipulation of the preserved specimens, it is clear that contraction of the *erectores dorsales I* abducts the gustatory stalk, causing it to dig into the substrate (Fig. 9). Adduction is accomplished by contraction of the *depressores dorsales I*. Since *inclinatores dorsales radiales I* are inserted at the mid-length of the first pterygiophore, their contractions would laterally abduct this bone and further displace the entire gustatory stalk laterally. This would cause the abducted gustatory stalk to dig further into or out of the substrate.

As a result of these rearrangements in the musculoskeletal system of the gustatory stalk, abduction and adduction of the organ involve its displacement not only in the anteroposterior axis but also in the lateral plane. In other fishes, the lateral displacement of the dorsal-fin rays is performed by the *inclinatores dorsales*. It is therefore not surprising that this muscle is missing in the gustatory stalk.

When the gustatory stalk is pulled out of its housing groove, the first regions to contact the substrate are the conical protuberance of the globular head and the anterior face of the main axis. These structures act like a plow or probe to penetrate and open space in the soft substrate. Accordingly, taste buds and microridges are relatively few in the main axis and completely absent in the conical protuberance, indicating that these regions play a more important role in penetrating the substrate than in tasting the environment. In addition, the swollen outer region of the rugged flap is completely perforated with multiple dimples that might be acting as a mucus storage system and therefore reducing friction while the gustatory stalk is in motion. Despite the physiological functions of mucus on the skin surface of fishes, it has mechanical implications such as reducing the percentage of which the skin is exposed to the environment and lubricating the outer surface of the skin, then protecting the fish's skin from any external force, including friction ^39^. Once inside the substrate, the taste buds of the gustatory stalk are in contact with the chemical substances released by potential buried prey. The taste buds and microridges are more densely distributed and better developed on the fimbriae and marginal region of the rugged flap. These are the most delicate structures of the gustatory stalk and are likely to play the most important role in chemical detection. All this finding corroborates the presence of dorsal-fin modifications with non-visual feeding habits of these flatfishes.

## Material and methods

This study was conducted under approval of the Animal Care and Use Committee (ACUC) of the Instituto de Biociências, Universidade de São Paulo to A. Datovo (Project #226/2015; CIAEP #01.0165.2014). Specimen sampling employed only ethanol-preserved specimens deposited in museums and did not involve animal experimentation or fossil examination. Two specimens of *Oncopterus darwinii* (MZUSP 72,703; MZUSP 10,030) were double stained following Datovo and Bockmann ^46^ to preserve soft tissues and allow the integrated view of bones, muscles, and nerves. One *O. darwinii* (MZUSP 72,660) specimen was examined under SEM in search of taste buds on the dorsal-fin rays. Image acquisition follows Presti, et al. ^47^. Digital bidimensional color illustrations were produced with a Wacom Intuos Comic pen tablet. Outlines were generated in Adobe Illustrator CC 2015 whereas shading and coloring were done in Adobe Photoshop CC 2015. SEM images were obtained from a LEO 440 Zeiss in which the tissues samples were dried to the critical point (Critical point dryer CPD 030 BAL-TEC) and then metalized posteriorly (Sputter Coater SCD 050 BAL-TEC). X-ray microcomputed tomography (μCT-scan) images were obtained from a microtomography Phoenix v|tome|x m microfocus of the General Electric Company. The image reconstructions were done by Phoenix datos|× 2 reconstruction; GE Sensing and Inspection Technologies GmbH and edited via VG Studio Max version 2.2.3.69611 64bits, Volume Graphics GmbH. Anatomical nomenclature follows Presti, et al. ^47^ and Voronina and Dias de Astarloa ^48^ for osteology, Winterbottom ^24^ for myology, Freihofer ^13^ for neurology, and Reutter, et al. ^14^ for taste buds. Comparative material: Achiridae: *Achirus declivis* (MZUSP 116,278), *Hypoclinemus mentalis* (MZUSP 101,594); Achiropsettidae: *Mancopsetta maculata* (USNM 362,528); Bothidae: *Bothus lunatus* (MZUSP 71,750), *Monolene antillarum* (MZUSP 72,275); Citharidae: *Citharus linguatula* (USNM 236,123); Cyclopsettidae: *Etropus crossotus* (MZUSP 118,902), *Cyclopsetta chiittendeni* (MZUSP 72,121); Cynoglossidae: *Symphurus jenynsi* (MZUSP 12,878); Paralichthodidae: *Paralichthodes algoensis* (SAIAB 118,830); Paralichthyidae: *Paralichthys isosceles* (MZUSP 91,684); Psettodidae: *Psettodes erumei* (MZUSP 63,360); Rhombosoleidae: *Peltorhamphus novaezeelandiae* (USNM 410,298); *Rhombosolea tapirina* (USNM 214,726); *Ammotretis rostratus* (USNM 282,708); Soleidae: *Brachirus orientallis* (MZUSP 63,355); *Solea vulgaris* (USNM 39,419).

## Data Availability

All data generated or analyzed during this study are included in this published article.
